# Umbilical Vessel Aneurysm Presenting a Large Placental Cyst: A Unique Case and Literature Review

**DOI:** 10.1177/10935266251352897

**Published:** 2025-07-02

**Authors:** Veronique Schiffer, Ina Thijs, Salwan Al-Nasiry, Stijn van Teeffelen, Carmen Severens-Rijvers

**Affiliations:** 1 Department of Gynaecology and Obstetrics, Maastricht University Medical Center, Maastricht, The Netherlands; 2 GROW, School for Oncology and Developmental Biology, Maastricht University, Maastricht, The Netherlands; 3 Department of Pathology, Maastricht University Medical Center, Maastricht, The Netherlands

**Keywords:** placenta, microscopy, fetal, neonatal, pediatric, soft tissue

## Abstract

We present a unique case of a 33-year-old gravida that was referred to our hospital with an umbilical vessel aneurysm presenting as a large placental cyst on ultrasound. Although the 20-week anomaly scan showed no structural abnormalities, routine fetal biometry scanning at 30 weeks of gestation revealed an abnormal placental cystic structure, located subchorionic under the umbilical cord insertion. Given the uncertainty of the origin of the structure’s origin and its unpredictable evolution with possible adverse effect on the fetus, a cesarean section was performed delivering a healthy baby. Histopathological examination of the placenta showed an aneurysmal vein with thinning of the vessel wall and fragmented smooth muscle. Umbilical cord aneurysm represents an exceptionally rare placental anomaly, with umbilical vein aneurysms being associated with variable fetal mortality rates, ranging from those observed in uncomplicated pregnancies to 82% in documented cases. Therefore, a multidisciplinary approach is essential to optimize fetal outcomes.

## Introduction

This is a unique case of an umbilical vessel aneurysm that was initially identified on an ultrasound scan as a placental cyst. Sonographic differentiation between a placental cyst and an umbilical vessel aneurysm is challenging and little is known about the differentiation, resulting in complex clinical decision-making regarding timing of labor and the method and frequency of fetal monitoring.

## Case Report

A 33-year-old woman, gravida 2 para 1, was referred to our academic hospital at 30 weeks and 5 days of gestation. Her previous pregnancy was uncomplicated, ending in a normal term vaginal delivery at 40 weeks and 2 days of gestation and a healthy son of 3360 g. Her current pregnancy was also uncomplicated and therefore prenatal care was given by a midwife. The non-invasive prenatal test (NIPT) and 20-week anomaly scan showed no structural abnormalities. She did not experience any blood loss and there was no traumatic event during this pregnancy. She had no other symptoms.

**Figure 1.**
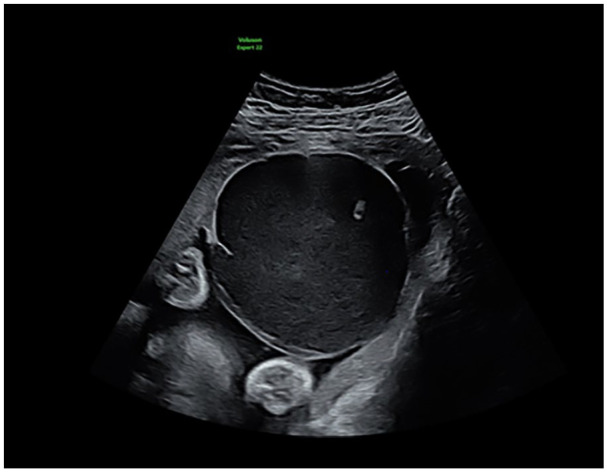
Large cystic placental structure (7.3 cm × 7.3 cm × 10.7 cm) located subchorionic under the insertion of the umbilical cord.

**Figure 2.**
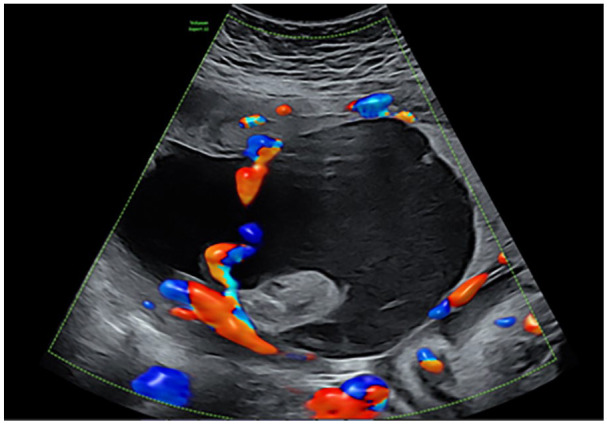
Large cystic placenta structure using Color Doppler, showing no internal vascularity within the structure itself, however abundant blood vessels around the cyst.

The patient was referred to our hospital because of an abnormal placental structure during routine fetal biometry scanning at 30 weeks of gestation. Ultrasound showed a large cystic placental structure (7.3 cm × 7.3 cm × 10.7 cm) located subchorionic under the insertion of the umbilical cord (Figure 1). There was a hyperechoic part visible within this structure and low-velocity venous blood flow within. Color Doppler showed no internal vascularity within the structure itself, however there were abundant blood vessels around it (Figure 2). Advanced fetal scan showed no structural abnormalities, a normal amount of amniotic fluid, and mild growth restriction; estimated fetal growth of 1347 g at the sixth percentile. Doppler measurement of the umbilical artery was normal, while the middle cerebral artery showed an elevated maximum velocity (*V*_max_) of 1.585 MoM. No other signs of anemia were seen. Fetal cell count in the maternal blood was negative.

The patient was admitted to our ward. A repeat scan the next day showed the same dimensions of the placental structure and at that time a normal *V*_max_ of the middle cerebral artery. Fetal monitoring with cardiotocography (CTG) was normal, the patient felt normal fetal movements, and did not report any symptoms.

However, given the uncertainty of the origin of the structure, its unpredictable evolution and possible adverse effect on the fetus, we decided to perform a cesarean section at 31 weeks and 1 day of gestation, after completion of the antenatal corticosteroid therapy. A healthy, pink-colored boy was born with Apgar score of 6/8/9 and birthweight of 1410 g (percentile 5–10). The umbilical cord was cut 1 minute after birth. We did not perform milking of the umbilical cord. Obtaining umbilical cord arterial and venous pH samples was not possible due to constriction of the umbilical vessels.

There was no indication for acute blood transfusion, however, delayed blood transfusion was needed 1 hour after birth, given his blood results (hemoglobin = 5.4 mmol/L, platelets = 76 × 10^9^/L, and reticulocytes = 52 × 10^9^/L [relative], and 112 × 10^9^/L [absolute]) and a more pale skin color. After blood transfusion, the boy was in good clinical condition. The placenta was manually removed during cesarean section and send to the Department of Pathology.

**Figure 3.**
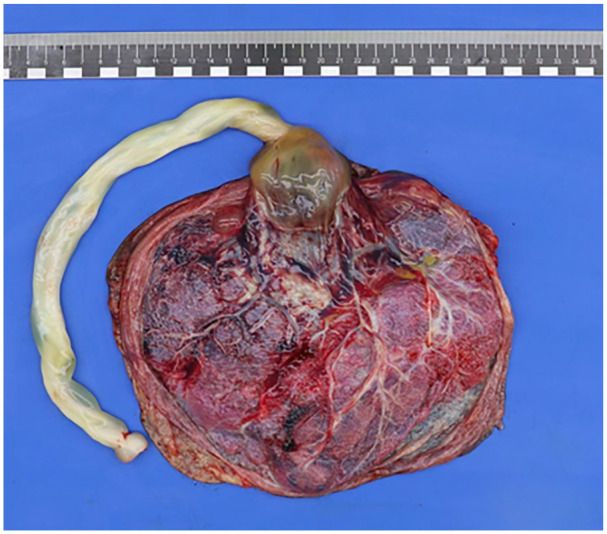
Macroscopic overview of fetal surface of the placenta, showing a remarkable, partly ruptured large cyst-like lesion at the base of the umbilical cord with measurements of 5.0 cm × 6.0 cm × 4.0 cm.

**Figure 4.**
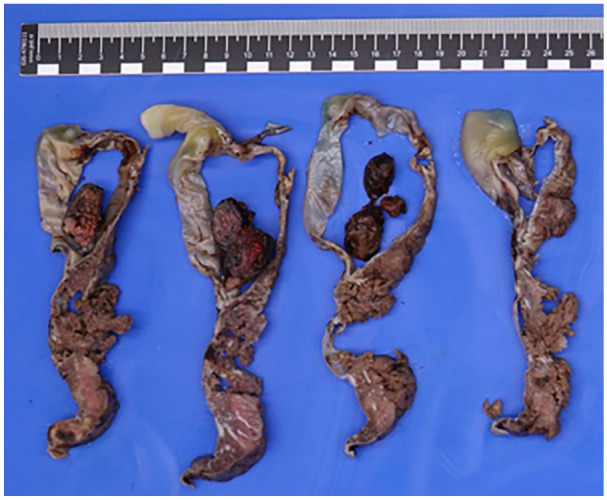
Umbilical cord with cyst-like lesion after formalin fixation, showing an organized blood clot on cut section.

Postpartum pathological examination of the placenta showed a peripheral insertion of the umbilical cord. The umbilical cord had a length of 28.0 cm and a diameter of 2.0 cm after formalin fixation and showed hypocoiling (coilings index 0.04). There were 3 vessels. The membranes were complete, translucent, and showed a normal peripheral implantation. The placental disc had a weight of 398 g (83 percentile) and measured 19.0 cm × 15.5 cm × 2.0 cm. On the fetal surface there was a remarkable large cyst-like lesion at the base of the umbilical cord with measurements of 5.0 cm × 6.0 cm × 4.0 cm (Figure 3), which was partly ruptured upon arrival at the Pathology department, probably resulting in smaller dimensions than measured on ultrasonography. After formalin fixation, there was an organized blood clot on cut section (Figure 4). Furthermore, there was a second cyst visible with a diameter of 2.0 cm in the chorionic plate. Histologically, the umbilical cord showed a normal composition of 2 umbilical arteries and 1 umbilical vein surrounded by Wharton’s jelly, without signs of thrombi or inflammation. However, the cystic structure at the base of the umbilical cord showed an aneurysmal vein with thinning of the vessel wall and fragmented smooth muscle. Further analysis of the placenta showed organized thrombi in the fetal blood vessels, leading to several foci of avascular villi (≥45 avascular villi, consistent with high grade fetal vascular malperfusion), chorangiosis, and increased nucleated red blood cells.

## Discussion

Sonographic differentiation between a placental cyst or an umbilical vessel aneurysm is challenging. Placental cysts are simple cystic lesions that develop in relation to the placenta. Their estimated prevalence is at 2–20% of all pregnancies, however evaluating prior experiences with cystic masses arising from the placenta is difficult given the variable terminology that has been used in the literature.^1^ Placental cysts can be categorized by location: within the placental tissue or underneath the fetal plate (subchorionic cysts). Location of the cysts close to the umbilical cord insertion have been noted previously, in which one of the differential diagnosis should be a subamniotic hemorrhage.^2^

In contrast, umbilical (vein or artery) aneurysm is a rare prenatal diagnosis with limited literature available regarding management. Previous literature shows wide variation regarding increased risk of fetal anomalies or adverse perinatal outcomes, in which the reported incidence of fetal malformations is highest in umbilical vein aneurysms (35%). A discrepancy exists within the literature, with studies reporting variable fetal mortality rates ranging from those comparable to uncomplicated pregnancies to 82% in documented cases.^3,4^ The cystic appearance of an umbilical aneurysm during ultrasonography can potentially lead to a misdiagnosis.

Umbilical vessel aneurysms may arise due to the inherent weakness of the umbilical arteries and veins at their insertion on the fetal plate, where Wharton’s jelly is relatively less abundant. This structural vulnerability, combined with increasing fetal cardiac output and elevated intravascular pressure, can lead to aneurysm formation in areas with greater elasticity where Wharton’s jelly is absent. The formation of large-sized aneurysms could lead to compression of surrounding umbilical vessels and vascular thrombosis following altered blood flow. The disruption of normal blood flow may also lead to endothelial damage, stasis of blood flow and activation of the coagulation cascade.^5^ In our case, we indeed found signs of high grade fetal vascular malperfusion. Furthermore, the placenta showed chorangiosis and increased nucleated red blood cells, indicative of chronic hypoxia.

Umbilical vessel aneurysm is a rare finding with a prevalence of 0.4–1.1 per 1000 and accounts for approximately 4% of umbilical vein malformations. The presence of high grade fetal vascular malperfusion due to the umbilical cord aneurysm has severe clinical implications whereas it is associated with fetal demise in >40% of cases.^5^ Furthermore, umbilical cord aneurysm is associated with fetal hypoxia and intrauterine growth restriction.^5,6^ The thinning of the vascular wall and reduction of Wharton’s jelly has a potential risk of aneurysm rupture, both intra-uterine or during labor, posing a significant threat of fetal death.

A brief literature review of all English-language articles reporting cases of umbilical vein aneurysm revealed that most publications consist of case reports (see *Supplemental Table 1*). Some case-reports were non-accessible (n = 8) and cases involving intra-abdominal pathology were excluded from this review (n = 8). The maximal diameter of the reported umbilical vein aneurysms ranged from 1.9 to 4 cm, with detection occurring between 21 and 34 weeks of gestation via ultrasound.^7,8^ Two case reports documented the delivery of healthy neonates at 30 + 3 and 35 weeks of gestation, both via caesarean section.^3,9^ Conversely, 1 case report described a stillbirth at 41 weeks of gestation,^8^ while another documented pregnancy termination due to concurrent congenital anomalies.^7^ Furthermore, umbilical cord aneurysm was associated with additional placental abnormalities and demonstrated a correlation with multiple congenital malformations. Overall, these studies show a wide variability in outcomes related to fetal anomalies and perinatal morbidity, emphasizing that there is a need for urgent referral to a tertiary center with expertise in placental pathology. We believe obstetric care should include detailed umbilical cord scanning during routine third trimester scans. As previously described, a multidisciplinary approach between fetal medicine specialists, neonatologists, and placenta pathologists is needed in case of a (suspected) umbilical vessel aneurysm weighing up the benefit of early delivery to avoid fetal death against the disadvantages of premature delivery. When the umbilical aneurysm is detected before 34 weeks of gestation, a course of antenatal corticosteroids to enhance lung maturity is advised. Furthermore, to prevent any lethal damage to the aneurysm caused by contractions, a cesarean section would be the indicated method of delivery.

## Supplemental Material

sj-docx-1-pdp-10.1177_10935266251352897 – Supplemental material for Umbilical Vessel Aneurysm Presenting a Large Placental Cyst: A Unique Case and Literature Review Supplemental material, sj-docx-1-pdp-10.1177_10935266251352897 for Umbilical Vessel Aneurysm Presenting a Large Placental Cyst: A Unique Case and Literature Review by Veronique Schiffer, Ina Thijs, Salwan Al-Nasiry, Stijn van Teeffelen and Carmen Severens-Rijvers in Pediatric and Developmental Pathology
