# Supplementary material for: Umbilical Vessel Aneurysm Presenting a Large Placental Cyst: A Unique Case and Literature Review
Source: Pediatr Dev Pathol. 2025 Jul 2;28(6):495–8. doi: 10.1177/10935266251352897 (PMC12589657; doi:10.1177/10935266251352897)
Supplement: sj-docx-1-pdp-10.1177_10935266251352897 – Supplemental material for Umbilical Vessel Aneurysm Presenting a Large Placental Cyst: A Unique Case and Literature Review [file sj-docx-1-pdp-10.1177_10935266251352897.docx]

**SUPPLEMENTAL
Table 1.** Overview of literature.

| **Author + title + year** | **Maternal age, gravida/para, gestational age at presentation** | **Measurements and location of aneurysm** | **Corticosteroids therapy?** | **Clinical outcome** | **Histopathological outcome** |
| --- | --- | --- | --- | --- | --- |
| Akar et al, 2012 | Exclusion: Fetal umbilical vein varix intra-abdominal, not of the umbilical cord. | | | | |
| Babay et al, 1996 | No access. | | | | |
| Berg et al, 2001 | 27-year old woman, gravida 1.  34 weeks gestation with suspected IUGR and oligohydramnios. Routine prenatal laboratory studies were normal.   Patient elected to terminate the pregnancy | 1.8 x 1.8 x 1.9 cm on ultrasound.  Pathological examination aneurysm of the umbilical cord in the distal third (closest to the fetus) and an arteriovenous fistula connecting two dilated vessels. | No | Patient elected to terminate the pregnancy.  Fetal blood sampling and karyotyping revealed trisomy 18 (47, XY+18). | Fetus: multiple malformations of the face, hands, feet.   Aneurysm of the umbilical cord in the distal third (closest to fetus) and an arteriovenous fistula connecting two dilated vessels.  Strongly dilated umbilical vein, one moderately dilated artery and a second normal artery. Vessel walls showed calcification of the intima and a thinned media. |
| Bhallis et al, 1997 | Exclusion: intra-abdominal varix of umbilical vein | | | | |
| Campbell et al, 2021 | 38 year, G2P1, 30 weeks and 3 days.  Maternal diabetes type II, an increase risk for trisomy 21 on first trimester screen. | 3-4 cm, in the free-floating cord. | Yes, two doses of betamethasone and a magnesium sulfate infusion for fetal neuroprotection. | Uncomplicated repeat cesarean section. Boy, 1320 gram (percentile <1). Platelet transfusion. Maternal HELLP syndrome postoperatively. | Uneven villous maturation, fetal thrombotic vasculopathy with endothelial cushion lesions, hemorrhagic endovasculatitis and focal acute deciduitis. |
| Cruise et al, 2002 | 30 year, G4P3, 24 weeks and 1 day. | 4 x 6 cm on ultrasound scan, cystic structure. | No. | Stillborn. Fetus was affected by Klippel-Trenaunay-Weber syndrome (abnormal right leg, buttocks, prostate, small area within the brain). | Trombosed venous dilatation of the umbilical cord, 5x 11cm. Twisting of the cord at each side of the varix was noted. |
| Deront-Bourdin et al, 2014 | No access, article in French. | | | | |
| Doehrman et al, 2014 | Exclusion: umbilical artery aneurysm | | | | |
| Estroff et al, 1992 | No access. | | | | |
| Hoon Lee et al, 2014 | 28 year, G2P0, 34 weeks | Umbilical cord 50mm in diameter at 34 weeks.  Umbilical cord 80 mm in diameter at 35 weeks. | ? | Cesarean section at 35 weeks of gestation. Boy, 2820 gram, Apgar 3 and 6.  Mild anemia in the baby’s laboratory test. Karyotyping was not conducted. | Dilated vein with an aneurysm and Wharton’s jelly filled with hemorrhagic fluid.  The placenta was grossly normal with a normal cord insertion. Umbilical cord was 35 in length, 60 mm in width (largest diameter) and with hemorrhagic fluid filled appearance. |
| Fung et al, 2005 | Exclusion: intra-abdominal varix of umbilical vein | | | | |
| Giannubilo et al, 2017 | No access. | | | | |
| Kostantinova et al, 1977 | No access. | | | | |
| Olog et al, 2011 | Exclusion: umbilical artery aneurysm | | | | |
| Panda et al, 2009 | No access. | | | | |
| Rahemtullah et al, 2001 | Exclusion: Article focused on intra-abdominal umbilical vein varices | | | | |
| Schröcksnadel et al, 1991 | 35 year, G4P3, 39 weeks and 6 days.  Uncomplicated pregnancy. | - | No | Stillbirth, female, 3130 gram, length 49 cm. | The insertion of the 70 cm long umbilical cord was marginal. At 4 cm distance from the placenta a 10 cm long bluish mass 4 cm in diameter was seen. The mass turned out to be a dilated umbilical vein (with a maximum diameter of 3 cm) and it was occulded by fresh thrombus with central fibrin deposition. |
| Sepulveda et al, 1998 | Exclusion: Varices of the intrafetal umbilical vein, not of the umbilical vein. | | | | |
| Shipp et al, 1995 | No access. | | | | |
| Siddiqi et al, 2012 | Exclusion: umbilical artery aneurysm | | | | |
| White et al, 1994 | 27 year, G3P2, 32 weeks and 2 days. | 2 cm distal to fetal abdomen, varix 2 x 2.3 cm (increasing over time). | Yes. | At 35 weeks of gestation, healthy girl, 2041 gram, cesarean section. | Moderate villous edema, dilated and tortuous umbilical vein varix and a recent partially occlusive umbilical arterial thrombosis adjacent to the varix. |
| Vandevijver et al, 2000 | 31 year old, primigravida.  At 21 weeks amenorrhea the fetus not the placenta and umbilical cord revealed abnormalities. | At approximately 10 cm from the chorionic plate, the umbilical cord showed a swelling of 4 cm in diameter. | No. | At 41 weeks, delivery of a lifeless daughter. No fetal heart beath after 5 h of labor. Patient has felt 30 min prior to admission fetal movements. | Fetus: Female stillborn weighed 3250 g (p50) and measured 56 cm (>p97). Autopsy showed petechial hemorrhages of the thymus, both lungs massive aspiration of meconium-containing amniotic fluid.  Strongly dilated umbilical vessel, on cut section an aneurysm of the umbilical vein. At the site of maximal dilatation there was abnormal thinning of the media with loss of muscle fibers and splitting of the elastic membrane. One of the umbilical arteries contained a fresh mural thrombus. Section of the placenta showed large, immature and hypervascular chorionic villi. |
| Zachariah et al, 2004 | No access. | | | | |
